# Prolonged parasite clearance in a Chinese splenectomized patient with falciparum malaria imported from Nigeria

**DOI:** 10.1186/s40249-017-0259-5

**Published:** 2017-04-04

**Authors:** Hong-Wei Zhang, San-Jin Li, Tao Hu, Yong-Min Yu, Cheng-Yun Yang, Rui-Min Zhou, Ying Liu, Jing Tang, Jing-Jing Wang, Xiu-Yun Wang, Yong-Xiang Sun, Zhan-Chun Feng, Bian-Li Xu

**Affiliations:** 1 Department of Parasite Disease Control and Prevention, Henan Center for Disease Control and Prevention, Zhengzhou, People’s Republic of China; 2 Department of Infectious Diseases, the 6th People’s Hospital of Zhengzhou, Zhengzhou, 450016 People’s Republic of China; 3 School of Medicine and Health Management, Tongji Medical College, Huazhong University of Science and Technology, Wuhan, 430030 Hubei People’s Republic of China

**Keywords:** Falciparum malaria, Artemisinin treatment, Prolonged parasite clearance, Splenectomy, China, Nigeria

## Abstract

**Background:**

The spleen plays a pivotal role in the rapid clearance of parasitized red blood cells in patients with falciparum malaria after artemisinin treatment. Prolonged parasite clearance can be found in patients who have had a splenectomy, or those with hemoglobin abnormalities and/or reduced immunity, which are all distinguishable from artemisinin resistance. This paper reports on a case of prolonged parasite clearance in a Chinese splenectomized patient with falciparum malaria imported from Nigeria.

**Case presentation:**

A 35-year-old Chinese male suffered 2 days of febrile illness after returning to Zhumadian city of Henan province from Nigeria on October 1, 2014. The main symptoms were febrile, including the highest axillary temperature of 40 °C, headache, and chills. A peripheral blood smear showed parasitemia (53 913 asexual parasites/μl) of *Plasmodium falciparum*. The patient had not used any chemoprophylaxis against malaria in Nigeria when he worked there as a construction worker between 2009 and 2014. The patient had three episodes of malaria in Nigeria and had a splenectomy due to a traffic accident 8 years ago from the time he was admitted to hospital. The patient was orally administrated a total of 320 mg/2.56 g dihydroartemisinin-piperaquine for 2 days and intravenously administrated a total of 3 000 mg artesunate for 18 days. The axillary temperature of the patient ranged between 37.0 and 37.7 °C from Day 0 to Day 3, and blood microscopy revealed falciparum malaria parasitemia (26 674 asexual parasites/μl) on Day 3. The patient was afebrile on Day 4, falciparum malaria parasitemia was continuously present and then gradually decreased on the next days, and was negative on Day 21. The patient was cured and left hospital on Day 24 after no plasmodium falciparum was found in the blood on Day 21 to Day 23. No mutation was found in the K13 propeller gene when compared with the PF3D7_1343700 K13 propeller gene reference sequence.

**Conclusions:**

This is the first reported case in China of prolonged parasite clearance in a splenectomized patient with imported falciparum malaria. Artemisinin resistance should be distinguished when prolonged parasite clearance is found in a malaria patient who has had splenectomy.

**Electronic supplementary material:**

The online version of this article (doi:10.1186/s40249-017-0259-5) contains supplementary material, which is available to authorized users.

## Multilingual abstract

Please see Additional file 1 for translations of abstract into five official working languages of the United Nations.

## Background

In humans, the spleen is the largest secondary immune organ in the body. Important functions of the spleen include removal of old and abnormal blood cells, removal of circulating pathogens, and facilitating the development of immune responses against *Plasmodium* [1]. It has been shown that the spleen plays an active role in the retention and removal of malaria-infected red blood cells (RBCs) from blood circulation, as well as a key role in the development of immune responses directed against parasites [2]. Parasite clearance rates are influenced by many factors, including insufficient artesunate and dihydroartemisinin levels in the blood, pharmacokinetics, pharmacodynamics, splenectomy, hemoglobin abnormalities, and reduced immunity, which may delay parasite clearance [3, 4]. The World Health Organization’s (WHO’s) classification of antimalarial drug efficacy (parasitemia on Day three with axillary temperature ≥ 37.5 °C) [5] may not apply to asplenic patients. This paper reports a case of prolonged parasite clearance in a splenectomized Chinese worker with falciparum malaria imported from Nigeria.

## Case presentation

A 35-year-old Chinese male was admitted to the 6^th^ People’s Hospital in Zhengzhou, China on October 6, 2014, because he had a fever with the highest axillary temperature of 40 °C, headache, and chills. Prior to admission, the patient had suffered 2 days of febrile illness after returning from Nigeria on October 1, 2014. The patient had not used chemoprophylaxis against malaria in Nigeria when he worked there as a construction worker between 2009 and 2014. The patient had three episodes of malaria and had been administrated quinine, but stopped treatment as soon as he was afebrile. The patient also had a splenectomy due to a traffic accident 8 years ago from the time he was admitted to hospital.

Upon admission, the patient’s axillary temperature was 37.2 °C, his pulse rate was 75 beats/min, his blood pressure was 93/79 mmHg, and his weight was 86 kg. Initial laboratory test results were almost normal except for a high C-reactive protein level of 35.8 mg/L and a low kalaemia level of 3.30 mmol/L (see Table 1). Giemsa-stained thick and thin blood films were microscopically examined and the peripheral blood smear showed parasitaemia of *Plasmodium falciparum*. Microscopy experts at the Henan Center for Disease Control and Prevention confirmed this result. The density of malarial parasites was 53 913 asexual parasites/μl on Day 0. A rapid diagnostic test (Wondfo Biotech Co., Ltd, China) was also positive for malarial antigen. DNA of *P. falciparum* was identified by nested polymerase chain reaction (PCR) amplification of the small-subunit ribosomal ribonucleic acid (18ssrRNA) genes. The K13 propeller gene was amplified using nested PCR and sequenced. No mutations were found when this gene was compared to the PF3D7_1343700 K13 propeller gene reference sequence retrieved from the National Center for Biotechnology Information (NCBI) database.

**Table 1:** Pre-treatment blood result

Variable	Value	Reference value
*Hematology*		
Leukocytes	6.93 × 10^9^/L	4–10 × 10^9^/L
Neutrophil	84.11%	45 ~ 77%
Lymphocyte	10.52%	20–40%
Monocyte	4.50%	3–8%
Eosinophil	0.60%	0.5–5%
Basophil	0.30%	0–1%
Erythrocytes	5.08 × 10^12^/L	3.5–5.5 × 10^12^/L
Hemoglobin	155 g/L	110–160 g/L
Hematocrit	46%	36–50%
Platelets	192 × 10^9^/L	100–300 × 10^9^/L
HCT	46.40	36.6–50%
MCV	89.70	86–100 fl
MCH	30.80	26–31 pg
MCHC	343.00	310–370 g/L
*Blood chemistry*		
AST	18 IU/L	<40
ALT	40 IU/L	<40
LDH	151 IU/L	110–240
γ-GTP	47 IU/L	<40
Total bilirubin	14.2 μmol/L	3.0–21.0
BUN	4.5 mmol/L	3.2–7.1
Creatinine	91 μmol/L	44–133
Uric acid	282 μmol/L	201–430
Sodium	134 mmol/L	135–145
Potassium	3.30 mmol/L	3.5–5.5
Chloride	97 mmol/L	98–110
CRP	35.8 mg/L	0.10–8.20

*Abbreviations*: *ALT* alanine aminotransferase, *AST* aspartate aminotransferase, *BUN* blood urea nitrogen, *CRP* C-reactive protein, *HCT* Hematocrit, *LDH* lactic dehydrogenase, *MCH* mean corpuscular hemoglobin, *MCHC* mean corpuscular hemoglobin concentration, *MCV* erythrocyte mean corpuscular volume, *γ-GTP* gamma-glutamyl transpeptidase

The patient was intravenously administered artesunate (120 mg first time; then 60 mg every six hours) for 4 days and intravenously treated with the antibiotic ceftriaxone (2.0 g per day) for 4 days. After 72 hours of treatment, the axillary temperature of the patient was 37.4 °C and the density of falciparum parasites was 26 674 asexual parasites/μl (see Table 2). The patient was continuously intravenously administered artesunate (60 mg every 8 h for 9 days; 60 mg every 12 h for 1 day; 60 mg every day for 4 days) with antibiotic ceftriaxone of 2.0 g per day for 14 days. The patient was afebrile on Day 4 but blood microscopy revealed the continuous presence of parasitaemia with densities between 16 133 and 34 356 asexual parasites/μl. The patient was orally administrated dihydroartemisinin-piperaquine (40 mg/0.32 g per tablet, two tablets twice per day) for 2 days from Day 10. The parasitemia declined slowly and morphology of the parasites in the blood films, with pyknotic nuclei and shrunken cytoplasm, suggested that they were dead. Falciparum parasitaemia was negative on Day 21. The patient was cured and left hospital on Day 24 after no plasmodium falciparum was found in the blood on Day 21 to Day 23.

**Table 2:** Parasite density and body temperature of patient during hospital stay

Day	Temperature (°C)	Parasite density (asexual parasites/μl)
0	37.2	53 913
1	37.0	30 581
2	37.7	30 545
3	37.4	26 674
4	36.5	34 356
5	36.6	31 701
6	36.2	23 896
7	36.5	25 316
8	37.2	21 982
9	37.5	16 133
10	37.5	19 847
11	36.5	–
12	37.2	16 434
13	36.3	–
14	36.2	2 962
15	36.3	–
16	36.2	3 211
17	36.3	–
18	36.7	2 688
19	35.9	–
20	36.7	364
21	36.5	0
22	36.0	0
23	36.4	0

## Discussion

Malaria had a wide geographical distribution in China but this has decreased dramatically due to the strengthening of malaria control measures in recent decades, especially in the Yunnan and Hainan provinces, and Huanghuai region [6–8]. No locally transmitted malaria cases have been reported since 2012, but imported malaria has increased remarkably in Henan, which may cause secondary spread and antimalarial drug resistance. In 2014, 216 imported malaria cases were reported, which was 24 times higher than the nine imported cases in 2005 in this province [9]. With increasing travel and migration, imported malaria has become a major public health challenge [10–12] and poses a severe threat to the malaria elimination program in China [13]. The WHO currently recommends artemisinin-based combination therapies (ACTs) as the first-line treatment for uncomplicated *P. falciparum* malaria [14], and oral dihydroartemisinin-piperaquine tablets are most commonly used in China [15]. Typically, a 3-day treatment course with an ACT reduces parasite densities and 95% of patients’ malaria blood slide test results are negative 48 h after treatment [16]. The emergence of artemisinin resistance, evidenced by delayed parasite clearance after artemisinin treatment, poses a serious threat to the global control of malaria. Artemisinin resistance is characterized by a reduced susceptibility of the ring stage of parasite development. It was first documented in western Cambodia [17, 18] and is now prevalent across an expanding area of Southeast Asia, including in Cambodia [19–21], Thailand [22, 23], and the Thai-Myanmar border [24].

Over the past decade, African countries have transitioned from using chloroquine or sulfadoxine-pyrimethamine to ACTs as a first-line policy for treating uncomplicated malaria [25]. Although ACT-resistant malaria is rare in Africa, there is a historical precedence of resistance to antimalarial medicine emerging in Asia and spreading to Africa [26]. Three cases of early treatment failure due to possible ACT-resistant *P. falciparum* malaria have been reported in Nigeria [27]. There has also been a case of possible artemether-lumefantrine treatment failure in an Italian traveler with uncomplicated *P. falciparum* malaria imported from the Democratic Republic of the Congo [28]. Routine monitoring is needed to ensure that the recommended first-line ACTs are effective for timely changes in national treatment policies and for the early detection of artemisinin resistance.

Prolonged parasite clearance is the basis for the current WHO working definition of artemisinin resistance [5] but it may not applicable for patients who have had a splenectomy [29–31]. In the case reported in this paper, blood microscopy revealed that falciparum parasitemia was continuously present and was negative on Day 20 after artemisinin treatment. According to the WHO classification of antimalarial drug efficacy (parasitemia on Day three with axillary temperature ≥ 37.5 °C) [5], the patient was considered an early treatment failure for the first time in China. Actually, it is caused by splenectomy but not artemisinin resistance.

The spleen is responsible for initiating immune reactions to blood-borne antigens and for filtering the blood of foreign material, and old or damaged RBCs. It plays a crucial role in the defense against infections with viruses, bacteria, fungi, and parasites [32]. The spleen plays a pivotal role in the rapid clearance of parasitized RBCs in patients with falciparum malaria after artemisinin treatment, and parasite clearance after artesunate treatment can be markedly prolonged in falciparum malaria patients who have had a splenectomy [33]. A previous study showed that *Plasmodium*-infected erythrocytes lose their normal deformability and become susceptible to splenic filtration, and splenic clearance of erythrocytes is enhanced by antimalarial therapy and splenomegaly [34]. The intact splenic function is of utmost importance for the host’s defense capacity against *Plasmodium* spp., not only because it limits the acute infection through the removal of parasites from the blood stream, but also because it modulates parasite antigen expression on the surface of infected RBCs as well as cellular and humoral immune responses [35]. Other causes of immune suppression, such as cancer, may possibly reactivate latent malaria parasites [36]. Falciparum malaria infection is common in pregnant women and anemia is an important complication [37]. HIV infection may defects in macrophage phagocytosis, which diminish the spleen’s ability to clear infection, causes the rapid onset of cerebral malaria in children [38].

The delay in parasite clearance has also been reported in a splenectomized patient with *P. knowlesi* malaria [39]. In a prospective controlled study of 33 previously splenectomized Malawian adults, parasite densities of *P. falciparum* reached significantly higher levels, and mature parasite stages were more often seen in the peripheral blood of asplenic individuals [40]. In areas endemic for *P. falciparum*, fever and parasitemia are significantly more frequent in splenectomized patients than in spleen-intact patients [40–42]. Splenectomized patients are at high risk of developing a more severe and fatal disease, even if they acquired semi-immunity prior to the loss of splenic tissue [29, 33, 35, 43]. In this case, the patient’s main symptoms were fever and parasitemia. The parasitemia declined slowly following artemisinin treatment. The morphology of the parasites in the blood films, with pyknotic nuclei and shrunken cytoplasm, suggested that they were dead. Although drug-affected parasites have clearly abnormal shapes, it is not easy to distinguish live parasites from dead ones. Malaria parasites were observed in blood films until Day 21 after continuous artemisinin treatment. Chotivanich et al. reported that patients without a functional spleen who have prolonged parasite clearance presumably do not require additional antimalarial treatment [33].

Recently, a molecular marker of artemisinin resistance was identified. Mutations in K13 propeller domain were shown to be associated with delayed parasite clearance in vitro and in vivo [44, 45]. The detection of molecular mutations associated with K13 will greatly facilitate the tracking of artemisinin resistance as it emerges. In the case reported in this paper, prolonged parasite clearance was found after treatment with artesunate and ACT. The patient had a splenectomy due to a traffic accident 8 years ago from the time he was admitted to hospital and no mutation was found in the K13 propeller gene. Therefore, this case was finally identified as prolonged parasite clearance by splenectomy.

## Conclusion

This paper reports a case of prolonged parasite clearance in a splenectomized Chinese worker with falciparum malaria imported from Nigeria. Artemisinin resistance should be distinguished when prolonged parasite clearance is found in a malaria patient who has had splenectomy.

## Additional file

Additional file 1: Multilingual abstract in the five official working languages of the United Nations. (PDF 694 kb)

## Abbreviations

ACT: Artemisinin-based combination therapy
PCR: Polymerase chain reaction
RBC: Red blood cell
WHO: World Health Organization
