# Supplementary material for: Prolonged parasite clearance in a Chinese splenectomized patient with falciparum malaria imported from Nigeria
Source: Infect Dis Poverty. 2017 Apr 4;6:44. doi: 10.1186/s40249-017-0259-5 (PMC5379605; doi:10.1186/s40249-017-0259-5)
Supplement: Additional file 1: — Multilingual abstract in the five official working languages of the United Nations. (PDF 694 kb) [file 40249_2017_259_MOESM1_ESM.pdf]

Translation of the abstract into the five official working languages of the United Nations

التخلص طويل الأمد من الطفيليات في مريض طحال صيني مصاب أيضا بملاريا منجلية آتية من نيجيريا

هونج وي تشانج، سان لى جين تاو هو جين تاو، يونج مين يو تشنج يون يانج، وروي مين تشو يينج ليو جينج تانج، جينج جينج وانج، شيوى يون وانج يونج شيانج سون، زان- تشون فنج، بيان لي شو

ملخص

خلفية: يلعب الطحال دورا محوريا في التخليص السريع من خلايا الدم الحمراء المصابة بالطفيل في المرضى الذين يعانون من الملاريا المنجلية بعد العلاج بالأرتيميسينين. ويظهر التخلص طويل الأمد من الطفيليات في المرضى الذين استؤصل طحالهم، أو الذين لديهم تشوهات في الهيموجلوبين و / أو انخفاض المناعة، وكلها يمكن تمييزها من مقاومة الأرتيميسينين. هذه الورقة تقرير عن حالة التخلص من الطفيليات طويل الأمد في مريض طحال صيني مصاب أيضا بملاريا منجلية آتية من نيجيريا.

عرض الحالة: أصيب رجل صيني يبلغ من العمر 35 عاما لمدة يومين بحمى بعد عودته إلى مدينة جومادیان من مقاطعة خنان من نيجيريا في 1 أكتوبر 2014. الأعراض الرئيسية للحمى كانت ارتفاع درجة حرارة التي بلغت عن القياس من الإبط 40 درجة مئوية، والصداع وقشعريرة. وأظهرت عينة الدم الطرفية الإصابة بالطفيل (53913 طفيل لاجنسي / ميكرولتر) من المتصورة المنجلية. ولم يعط المريض أي وقاية كيميائية ضد الملاريا في نيجيريا عندما كان يعمل هناك عامل بناء بين عامي 2009 و2014. ومر المريض بثلاث أطوار من الملاريا في نيجيريا، وكان استئصال الطحال بسبب حادث مروري قبل ثماني سنوات حيث أودع المستشفى. أعطي المريض عن طريق الفم ما مجموعه 320 مجم / 2.56 جرام دي هيدروارتيميسينين/بيبيراكين لمدة يومين وأعطي عن طريق الوريد ما مجموعه 3000 مجم من الأرتيسونات لمدة 18 يوما. وتراوحت درجة الحرارة المقاسة من الإبط للمريض بين 37.0 و37.7 درجة مئوية من يوم 0 إلى اليوم الثالث، وكشف الفحص المجهري للدم عن وجود طفيل الملاريا المنجلية 26674 طفيل لاجنسي / ميكرولتر) في اليوم الثالث. وزالت الحمى عن المريض في اليوم الرابع مع بقاء طفيل الملاريا المنجلية الذي انخفض تدريجيا في الأيام التالية، وكان سلبيا في اليوم 21. شفي المريض وغادر المستشفى يوم 24 بعد أن تلاشت المتصورات المنجلية في الدم في اليوم 21 إلى اليوم 23. ولم يعثر على أي طفرة في الجين مروحي الشكل K13 بالمقارنة مع التسلسل المرجعي للجين مروحي الشكل K13 PF3D7_1343700.

الاستنتاجات: هذه هي أول حالة في الصين للتخلص طويل الأمد من الطفيليات في مريض طحال مصاب أيضا بالملاريا المنجلية من الخارج. يجب ملاحظة مقاومة الأرتيميسينين عند حدوثها في مريض ملاريا يخضع لعلاج طويل الأمد للتخلص من الطفيليات وتم استئصال طحاله.

Translated from English version into Arabic by Mahmoud Sami, through

TRANSLATORS
WITHOUT BORDERS

尼日利亚回国输入性恶性疟患者脾切除致疟原虫清除延迟

张红卫，李三景，胡桃，于永敏，杨成运，周瑞敏，刘颖，唐静，王静静，王秀云，孙永祥，冯占春，许汴利

摘要

引言：青蒿素治疗恶性疟患者时，脾脏在迅速清除红细胞内的疟原虫中起到关键作用。与青蒿素耐药不同，疟原虫清除延迟可见于脾切除、血红蛋白异常和免疫力低下患者。本文报道一例尼日利亚回国输入性恶性疟患者脾切除致疟原虫清除延迟。

病例介绍：患者，男，35 岁，于 2014 年 10 月 1 日从尼日利亚回到河南省驻马店市，以“发热 2 天”为主诉入院，最高体温 40°C，伴头痛，寒战。外周血恶性疟原虫密度 53,913 无性体/微升血。患者 2009 年-2014 年在尼日利亚做建筑工人，期间没有服药疟疾预防药，有 3 次疟疾发作。8 年前因交通事故行脾脏切除术。入院后给予口服总剂量 320mg/2.56g 双氢青蒿

素派喹片 2 天，后静脉注射总剂量 3000 mg 青蒿琥酯针治疗 18 天。入院后体温 37.0- 37.7°C，治疗第三天疟原虫密度 26,674 无性体/微升血。治疗第四天体温降至正常，原虫密度持续存在并逐渐下降，第 20 天疟原虫转阴，连续 3 天疟原虫阴性后出院。K13 基因检测未发现突变。
结论：国内首次报道输入性恶性疟患者脾切除致疟原虫清除延迟。疟疾患者疟原虫清除延迟时需要区别是脾切除所致还是青蒿素耐药。

Translated from English version into Chinese by Hong-Wei Zhang

Élimination parasitaire prolongée chez un patient chinois splenectomisé porteur d'un paludisme à *P. falciparum* importé du Nigeria

Hong-Wei Zhang, San-Jin Li, Tao Hu, Yong-Min Yu, Cheng-Yun Yang, Rui-Min Zhou, Ying Liu, Jing Tang, Jing-Jing Wang, Xiu-Yun Wang, Yong-Xiang Sun, Zhan-Chun Feng, Bian-Li Xu

RÉSUMÉ

Contexte : La rate joue un rôle essentiel dans l'élimination rapide des globules rouges parasités chez les patients infectés par le paludisme, après un traitement à l'artémisinine. Une élimination parasitaire prolongée peut s'observer chez des patients ayant subi une splénectomie ou présentant des anomalies de l'hémoglobine et/ou une immunité réduite ; toutes ces situations peuvent être distinguées de la résistance à l'artémisinine. Cet article rapporte un cas d'élimination parasitaire prolongée chez un patient chinois splenectomisé porteur d'une infection à *P. falciparum* importée du Nigeria.
Présentation du cas : Un homme chinois de 35 ans a souffert d'une maladie fébrile pendant deux après jours après être revenu du Nigeria à la ville de Zhumadian, dans la province chinoise du Henan, le 1er octobre 2014. Les symptômes principaux étaient de la fièvre, avec une température axillaire maximale de 40 °C, des céphalée et des frissons. Un frottis de sang périphérique a révélé la présence de *Plasmodium falciparum* (53 913 parasites asexués/µl). Le patient n'avait pas utilisé de chimioprophylaxie antipaludique pendant qu'il travaillait au Nigeria comme ouvrier du bâtiment, entre 2009 et 2014. Il avait eu trois épisodes palustres au Nigeria et avait subi une splénectomie à la suite d'un accident de la route, huit ans avant son hospitalisation. Le patient a reçu un total de 320 mg/2,56 g de dihydroartémisinine-pipéraquine par voie orale pendant deux jours et un total de 3000 mg d'artémusate par intraveineuse pendant 18 jours. Sa température axillaire s'est établie entre de 37,0 à 37,7 °C pendant les trois premiers jours et la microscopie sur frottis sanguin a révélé une parasitémie de 26 674 parasites asexués/µl au troisième jour. Le patient était afébrile au quatrième jour, le taux de *P. falciparum* était toujours positif et a diminué progressivement dans les jours qui ont suivi jusqu'à être négatif à J21. Le patient a été déclaré guéri et il est sorti de l'hôpital à J24, après des tests de parasitémie négatifs entre J21 et J23. Aucune mutation n'a été trouvée dans le gène K13 quand il a été comparé à la séquence de référence PF3D7_1343700 du gène *propeller* K13.
Conclusions : Ceci est le premier cas d'élimination parasitaire prolongée rapporté en Chine sur un patient splenectomisé porteur d'un paludisme importé. Il importe de faire la différence avec la résistance à l'artémisinine lorsqu'une élimination parasitaire prolongée est observée chez un patient splénectomisé.

Translated from English version into French by Suzanne Assenat, through

Длительное очищение от паразита в китайском splenectomized пациентом с тропической малярии, импортируемые из Нигерии

Hong-Wei Zhang, San-Jin Li, Tao Hu, Yong-Min Yu, Cheng-Yun Yang, Rui-Min Zhou, Ying Liu, Jing Tang, Jing-Jing Wang, Xiu-Yun Wang, Yong-Xiang Sun, Zhan-Chun Feng, Bian-Li Xu

РЕФЕРАТ

Фон: Селезенка играет важную роль в быстром оформлении паразитировали эритроцитов у больных с тропической малярией после лечения артемизинином. Длительное оформление паразит может быть найден у пациентов, перенесших спленэктомию, или те, с аномалиями гемоглобина и/или с снижением иммунитета, которые отличаются от устойчивости к артемизинину. Эта бумага сообщает о случае длительного оформления паразитирует в китайском splenectomized пациентом с тропической малярии, которая импортируется из Нигерии.

Презентация: 35-летний китайский мужчина страдал два дня лихорадочной заболеванией после возвращения в городе Чжумадянь провинции Хэнань из Нигерии на 1 октября 2014 года. Основными симптомами были лихорадка, в том числе самая высокая температура подмышечной 40 °C, головная боль и озноб. Периферический мазок крови показал паразитемии (53 913 бесполых паразитов/мкл) малярийного Плазмодия. Пациент не использовал любой химиопрофилактики против малярии в Нигерии, когда он работал там на стройке с 2009 по 2014. У пациента было три эпизода с малярией в Нигерии и спленэктомии вследствие дорожно-транспортного происшествия восемь лет назад от времени он был доставлен в больницу. Больному перорально вводят в общей сложности 320 мг/2.56 г dihydroartemisinin-piperaquine два дня и внутривенно вводят в общей сложности 3 000 мг артесуната в течение 18 дней. Температура подмышечной пациента колеблется между 37.0 и 37,7 °C от дня 0 до 3-х суток, а в крови при микроскопии выявляются тропической малярией паразитемии (26 674 бесполых паразитов/мкл) на 3 день. Пациент был afebrile на 4-й день, тропическая малярия паразитемии непрерывно и затем постепенно снижается в последующие дни, и была отрицательным на 21 день. Больной вылечился и покинул госпиталь на 24-й день после малярийного плазмодия был найден в крови на 21-й день на 23 дня. Никакая мутация была найдена в гене пропеллер K13 по сравнению с PF3D7_1343700 K13 ген пропеллер последовательность ссылок.

Заключение: Это первый случай в Китае длительного очищения от паразита у пациента splenectomized с завозной тропической малярией. Устойчивости к артемизинину следует отличать при длительном оформление паразит найден у больного малярией, который имел спленэктомии.

Translated from English version into Russian by Hao-Qi Zhang

Eliminación prolongada del parásito de malaria por falciparum, importada de Nigeria, en un paciente chino esplenectomizado

Hong-Wei Zhang, San-Jin Li, Tao Hu, Yong-Min Yu, Cheng-Yun Yang, Rui-Min Zhou, Ying Liu, Jing Tang, Jing-Jing Wang, Xiu-Yun Wang, Yong-Xiang Sun, Zhan-Chun Feng, Bian-Li Xu

RESUMEN

Antecedentes: El bazo desempeña un papel fundamental en la eliminación rápida de los glóbulos rojos parasitados en pacientes con malaria por falciparum después del tratamiento con artemisinina. La eliminación prolongada del parásito se puede encontrar en pacientes que han tenido una esplenectomía, o en aquellos con anomalías de la hemoglobina y/o inmunidad reducida, todos distinguibles de resistencia a la artemisinina. Este artículo informa sobre un caso de eliminación prolongada de parásitos de malaria por falciparum importada de Nigeria, en un paciente chino esplenectomizado.

Presentación del caso: Un hombre chino de 35 años de edad sufrió dos días de enfermedad febril después de regresar de Nigeria a la ciudad de Zhumadian en la provincia de Henan, el 1 de octubre de 2014. Los síntomas principales fueron febriles, incluyendo temperatura axilar más alta de 40 °C, dolor de cabeza y escalofríos.

Una frotis de sangre periférica mostró parasitemia (53 913 parásitos asexuales/µl) de Plasmodium falciparum. El paciente no había utilizado ninguna quimioprofilaxis contra la malaria en Nigeria cuando trabajó allí como trabajador de la construcción entre 2009 y 2014. El paciente tuvo tres episodios de malaria en Nigeria y una esplenectomía debido a un accidente de tráfico ocho años antes del momento en que fue ingresado en el hospital. El paciente recibió por vía oral un total de 320 mg/2,56 g de dihidroartemisinina-piperaquina durante dos días y se le administraron, por vía intravenosa, un total de 3000 mg de artesunato durante 18 días La temperatura axilar del paciente osciló entre 37,0 y 37 °C desde el día 0 hasta el día 3, y la microscopía de sangre reveló parasitemia de malaria por falciparum (26.674 parásitos asexuales/µl) el día 3. El paciente estuvo sin fiebre el día 4, la parasitemia de la malaria por falciparum estuvo presente continuamente y luego disminuyó gradualmente en los días siguientes y fue negativa en el día 21. El paciente se curó y dejó el hospital al día 24 después de que no se encontró plasmodium falciparum en la sangre desde el día 21 al día 23. No se encontró mutación en el gen propeller K13 cuando se compara con la secuencia de referencia del gen propeller K13 de PF3D7_1343700.

Conclusiones: Este es el primer caso reportado en China de eliminación parasitaria prolongada de malaria por falciparum, importada, en un paciente esplenectomizado. La resistencia a la artemisinina debe distinguirse cuando se encuentra una eliminación parasitaria prolongada en un paciente de malaria que ha tenido una esplenectomía.

Translated from English version into Spanish by patriciacassoni, through

TRANSLATORS
WITHOUT BORDERS
